# Anticoagulant use for the prevention of stroke in patients with atrial fibrillation: findings from a multi-payer analysis

**DOI:** 10.1186/1472-6963-14-329

**Published:** 2014-07-28

**Authors:** Kathleen Lang, Duygu Bozkaya, Aarti A Patel, Brian Macomson, Winnie Nelson, Gary Owens, Samir Mody, Jeff Schein, Joseph Menzin

**Affiliations:** 1 Boston Health Economics, Inc, Waltham, MA, USA; 2 Janssen Scientific Affairs, LLC, Raritan, NJ, USA; 3 Gary Owens Associates Inc, Ocean View, DE, USA

**Keywords:** Anticoagulant, Stroke, Prevention, Atrial fibrillation, Warfarin

## Abstract

**Background:**

Oral anticoagulation is recommended for stroke prevention in intermediate/high stroke risk atrial fibrillation (AF) patients. The objective of this study was to demonstrate the usefulness of analytic software tools for descriptive analyses of disease management in atrial AF; a secondary objective is to demonstrate patterns of potential anticoagulant undertreatment in AF.

**Methods:**

Retrospective data analyses were performed using the Anticoagulant Quality Improvement Analyzer (AQuIA), a software tool designed to analyze health plan data. Two-year data from five databases were analyzed: IMS LifeLink (IMS), MarketScan Commercial (MarketScanCommercial), MarketScan Medicare Supplemental (MarketScanMedicare), Clinformatics™ DataMart, a product of OptumInsight Life Sciences (Optum), and a Medicaid Database (Medicaid). Included patients were ≥ 18 years old with a new or existing diagnosis of AF. The first observed AF diagnosis constituted the index date, with patient outcomes assessed over a one year period. Key study measures included stroke risk level, anticoagulant use, and frequency of International Normalized Ratio (INR) monitoring.

**Results:**

High stroke risk (CHADS_2_ ≥ 2 points) was estimated in 54% (IMS), 22% (MarketScanCommercial), 64% (MarketscanMedicare), 42% (Optum) and 62% (Medicaid) of the total eligible population. Overall, 35%, 29%, 38%, 39% and 16% of all AF patients received an anticoagulant medication in IMS, MarketScanCommercial, MarketScanMedicare, Optum and Medicaid, respectively. Among patients at high risk for stroke, 19% to 51% received any anticoagulant.

**Conclusions:**

The AQuIA provided a consistent platform for analysis across multiple AF populations with varying baseline characteristics. Analyzer results show that many high-risk AF patients in selected commercial, Medicare-eligible, and Medicaid populations do not receive appropriate thromboprophylaxis, as recommended by treatment guidelines.

## Background

Atrial fibrillation (AF) is the most common sustained cardiac arrhythmia, diagnosed in approximately 1% of the general population [1]. AF is projected to affect over 7.5 million people in the United States (U.S.) by 2050 and poses a significant burden [2]. Among patients with AF without prophylaxis, the risk of stroke is 5 times higher than in persons free of the disease [3,4]. The estimated direct and indirect cost of stroke in 2008 was substantial ($34.3 billion) [3]. Furthermore, the mean individual lifetime cost of ischemic stroke was estimated to be $223,714 (2011 USD) [3,5].

Thromboprophylaxis with oral anticoagulants involving warfarin or other agents is the mainstay for stroke prevention, reducing the annual incidence of stroke in AF patients by more than 60% [6]. However, thromboprophylaxis is generally under-utilized among AF patients [7,8]. Boulanger et al. reported that, among Medicaid eligible patients who did not have contraindications to warfarin, claims for valve replacement procedures, or evidence that AF resulted from transient or reversible causes, 59% filled any prescriptions for warfarin following AF diagnosis [7]. Among patients from the REduction of Atherothrombosis for Continued Health (REACH) Registry, only 59% of the high risk patients with AF were treated with oral anticoagulants [8]. While contraindications may contribute to low rates of anticoagulation, recently released results from the Outcomes Registry for Better Informed Treatment of Atrial Fibrillation (ORBIT-AF) show that rates of anticoagulation as high as 80% among AF patients with high stroke risk (88% among patients without contraindications) are attainable [9].

The current health care environment places a strong emphasis on quality of care. In AF, quality is assessed in terms of provider adherence to three primary areas of stroke prevention [10]. These involve use of chronic anticoagulation therapy, the assessment of risk factors for thromboembolism and disease progression, and International Normalized Ratio (INR) monitoring. While patient registries, such as ORBIT-AF [9,11], have started to collect data on these measures among a large U.S. sample, assessment of quality measures from a health plan perspective would help decision makers monitor practice patterns and opportunities for improvement among a managed population. This study aims to demonstrate the usefulness of analytic software tools for the evaluation of AF disease management and resource utilization from Medicare, Medicaid, and commercial insurance perspectives. A secondary objective is to compare the quality measures generated from the analytic tool against current AF treatment guidelines [12] (e.g., patterns of potential anticoagulant under-treatment) for each payer type. For this purpose, a condition-specific software tool, the Anticoagulant Quality Improvement Analyzer (AQuIA), was created. The AQuIA provides a common analysis platform, which ensures that various population health data are evaluated in a consistent way by eliminating variations in outcome definitions and methodology, and focusing on understanding how findings vary across populations that differ based on age, comorbidities, and other factors. While previously published studies have used health plan data from a single payer perspective to evaluate the utilization of anticoagulants among patients with AF [13,14], our analysis adds to the literature by providing current estimates among patients from the Medicare, Medicaid, and commercial insurance perspectives.

## Methods

### Data sources

This study used five different anonymized, integrated databases including medical and pharmacy claims. Diagnoses and procedures were identified based on International Classification of Diseases Ninth Revision Clinical Modification (ICD-9-CM) and Current Procedural Terminology (CPT) codes from patients’ medical claims, while medication use was assessed based on National Drug Codes (NDC) from patients’ pharmacy claims.

The IMS LifeLink® Health Plan Claims Database is a commercial database consisting of approximately 55 million patients from over 75 managed care organizations across the U.S. and several million Medicare managed-care enrollees from four U.S. geographical regions. This database consists of two files, including a claims file and an eligibility file. The claims file contains details on: medical and pharmacy claims, including date of service, place of service, ICD-9-CM codes, CPT codes, physician specialty, NDCs, drug quantity dispensed, days supplied, charged and paid amounts, and copayments. The eligibility file includes monthly medical and pharmacy eligibility flags as well as patient demographic data.

The MarketScan® Commercial Claims and Encounter and Medicare Supplemental and Coordination of Benefits Databases are constructed from privately insured paid medical and prescription drug claims for approximately 30 million employees and their dependents (in 2010) [15]. “Commercial Claims and Encounter” and “Medicare Supplemental and Coordination of Benefits” are provided as separate databases. The MarketScan Medicare Database contains the healthcare experience of individuals with Medicare supplemental insurance paid by employers for approximately 3.42 million retirees (in 2010) [15]. Medical claims capture details regarding dates of service, place of service, physician specialty, up to four ICD-9-CM diagnosis codes, CPT codes, charges, and health plan payments (both the Medicare-paid and employer-paid supplemental amounts are included). Pharmacy claims include details on dispense date, NDCs, quantity of medication dispensed, days supplied, and health plan payments. Eligibility file contains details on monthly medical and pharmacy eligibility, age, sex, and geographical region for individuals who are present in the claims file.

Clinformatics™ DataMart, a product of OptumInsight Life Sciences, Inc. (Eden Prairie, MN) (Optum), consists of a commercially insured population from a diverse group of health plans in the United States including 30 million individuals (during 2002 to 2007) [16]. The medical and pharmacy claims files contain details on date of service, place of service, ICD-9-CM codes, CPT codes, provider type, NDCs, drug quantity dispensed, days supplied, charges, deductibles and copayments. The member file includes information on eligibility periods as well as patient demographic data.

The southern U.S. Medicaid program covers low-income or disabled individuals and consists of two files: a claims file, with details on medical and pharmacy utilization, including date of service, place of service, ICD-9-CM codes, CPT codes, physician specialty, NDCs, drug quantity dispensed, days supplied, and paid amounts; as well as an eligibility file, with details on monthly enrollment and patient demographics.

The most recent two-years of claims available from each database were used; IMS LifeLink Database (IMS)- including commercially insured claims, 07/2009-06/2011 MarketScan Commercial Database (MarketScanCommercial)- including commercially insured claims, 07/2009-06/2011; MarketScan Medicare Supplemental Database (MarketScanMedicare)- including employer-sponsored Medicare Supplemental plans only, 07/2009-06/2011; Clinformatics™ DataMart, a product of OptumInsight Life Sciences, Inc. (Eden Prairie, MN) (Optum)- including commercially insured claims, 04/2010-03/2012, and a Medicaid Database for a southern US state (Medicaid), 07/2008-06/2010. Given the large sample sizes in the IMS, MarketScanCommercial, MarketScanMedicare and Optum databases, a 10% random sample was selected from each of these, while the full Medicaid sample was used.

### Patient selection

Patients were included in the study if they were ≥18 years of age and had at least one primary or secondary diagnosis of AF, determined based on the ICD-9-CM code 427.31, within a two-year period.

The index date was defined as the date of the first AF diagnosis. Patients were followed over a period of one year after the index date (i.e., the study period). All demographic and outcomes data were evaluated during the study period. Additional criteria for continuous eligibility was not applied; however, patients who were Medicare and/or health maintenance organization (HMO) eligible anytime during the two-year period were excluded in the Medicaid database, in order to ensure the availability of all claims within this database.

### Study measures

Demographics (i.e., age and sex) of patients, stroke risk scores, comorbidities, use of anticoagulants, stroke related hospitalizations, frequency of INR tests and all-cause resource use were assessed during the one year period following the first occurrence of the diagnosis of AF.

The level of stroke risk (i.e., low, medium, high) was assessed using CHADS_2_ and CHA_2_DS_2_-VASc scores. For determination of the CHADS_2_ score, one point each was assigned for the presence of Congestive heart failure (CHF), Hypertension (HTN), Age ≥75 years, or diabetes (ICD-9-CM codes available in Additional file 1: Table S1). Two points were assigned for a history of stroke or transient ischemic attack (TIA). For determination of the CHA_2_DS_2_-VASc score [C = CHF/Left ventricular dysfunction (LVH), H = HTN, A = Age (≥75), D = Diabetes, S2 = Stroke/TIA, V = Vascular disease, A = Age 65–74, and Sc = Sex category], one point each was assigned for the presence of CHF/ LVH, HTN (systolic blood pressure >160 mmHg), age being 65–74 years, diabetes, vascular disease (coronary artery disease, heart attack, peripheral artery disease, aortic plaque) and sex category being female. Two points were assigned for the presence of each of the following factors: Age ≥75 and history of stroke, TIA, or thromboembolism. Patients with AF were subsequently assigned to one of the following categories based on their risk factors for stroke; low risk (0 points), moderate risk (1 point), or high risk (≥2 points).

The percentage of patients using anticoagulants was determined based on prescription claims with national drug codes (NDCs). Patients with at least one prescription claim for an anticoagulant medication were categorized as receiving treatment. The number and percentage of patients with a gap in anticoagulant therapy and the time to the first gap in anticoagulant therapy (i.e., the number of days from the start of an anticoagulant drug to the start of a gap in anticoagulant therapy defined as 60 days or more) were determined using pharmacy claims. Anticoagulant medical possession ratio (MPR) was calculated as follows: ([Days supply any anticoagulant] - [Last fill days supply])/([Last prescription fill date in data set] - [First prescription fill date]). In cases where the days supply of any two anticoagulant medications overlapped by more than 25% of their total supply, only the unique days were included in the numerator (i.e., overlapping prescriptions with the same date of service were not double counted).

Percentage of patients hospitalized for stroke was identified based on ICD-9-CM (primary code indicative of ischemic and/or hemorrhagic stroke) and CPT/UB-92 (uniform billing) codes (indicative of hospitalization or outpatient visits) from medical claims. Among patients hospitalized for stroke, outpatient use of anticoagulants was also determined. The number and percentage of patients with a bleeding event were identified based on inpatient hospitalizations associated with a primary ICD-9-CM code indicative of bleeding. Condition specific ICD-9-CM codes are provided in the Appendix (Additional file 2: Table S2, Additional file 3: Table S3).

INR frequency of occurrence was evaluated following warfarin use. The number of average unique INRs per month of warfarin treatment was calculated as number of unique INRs during treatment months divided by number of months taking warfarin (calculated based on dispense days and days supplied). The monthly INR quality ratio was calculated as the number of months with one or more INRs during months taking warfarin divided by the number of months taking warfarin. Ratios range from 0–1, with higher values representing better INR quality ratios. INRs reflect only those captured in claims data. If an INR was performed during a visit and not recorded on a claim, it is not captured in the tool.

The percentage of patients with an inpatient hospitalization, an ER visit and an outpatient visit were reported for the study period, as well as the mean number of visits per patient for each all-cause resource category.

### Data analyses

All analyses were descriptive in nature with no multivariate analyses performed. Categorical variables were summarized using counts and sample proportions. Mean values were reported for continuous measures. Analyses stratified by stroke risk level, anticoagulant treatment status and by age group were conducted.

SAS software (Version 9.3, SAS Institute, Cary, NC) was used for extracting medical/pharmacy claims and demographic information from all databases for AF patients, and for organizing this information so that the data were in the proper format to be utilized by the software tool, which carried out the analyses that produced the study outcomes. The software tool is condition-specific and Health Insurance Portability and Accountability Act–compliant, and enables the uploading of pharmacy and medical claims data via a simple point-and-click method to produce results for a series of predetermined and user-defined measures and to generate sample-specific reports. This study did not directly involve human subjects, and all study data were anonymized prior to being received; therefore, this study did not require ethical review or approval in order to be conducted.

## Results

### Demographic and clinical characteristics

**Figure 1:**
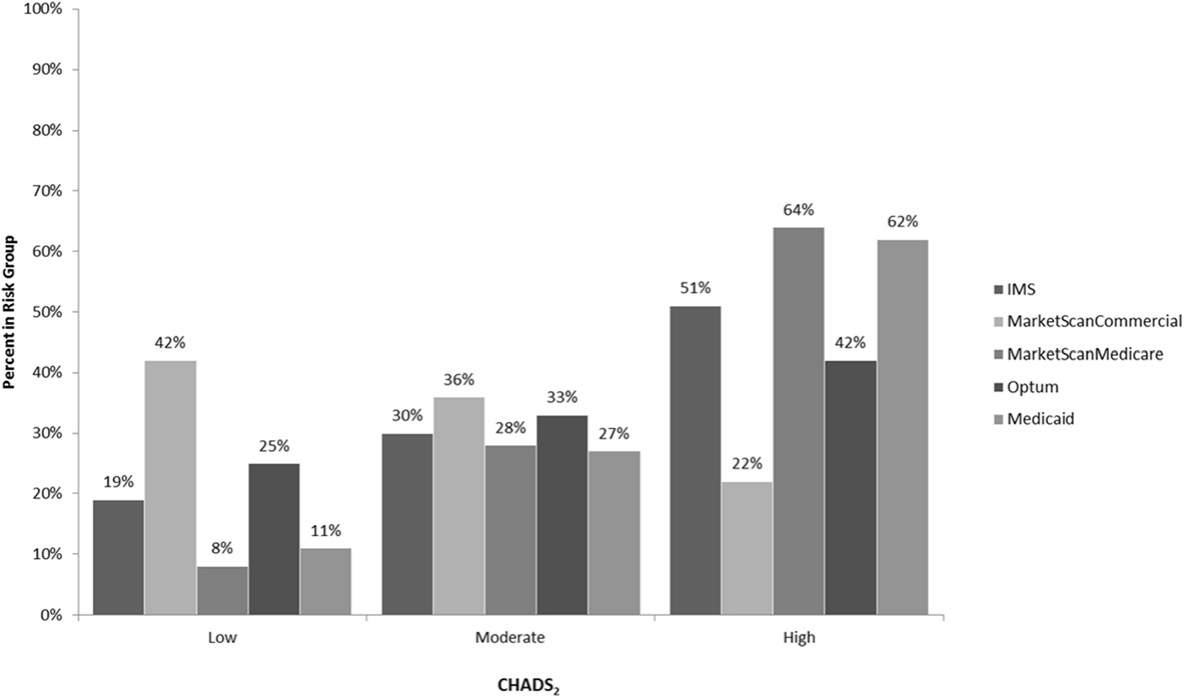
**Proportion of AF patients in each CHADS**_**2 **_**stroke risk level.**

**Figure 2:**
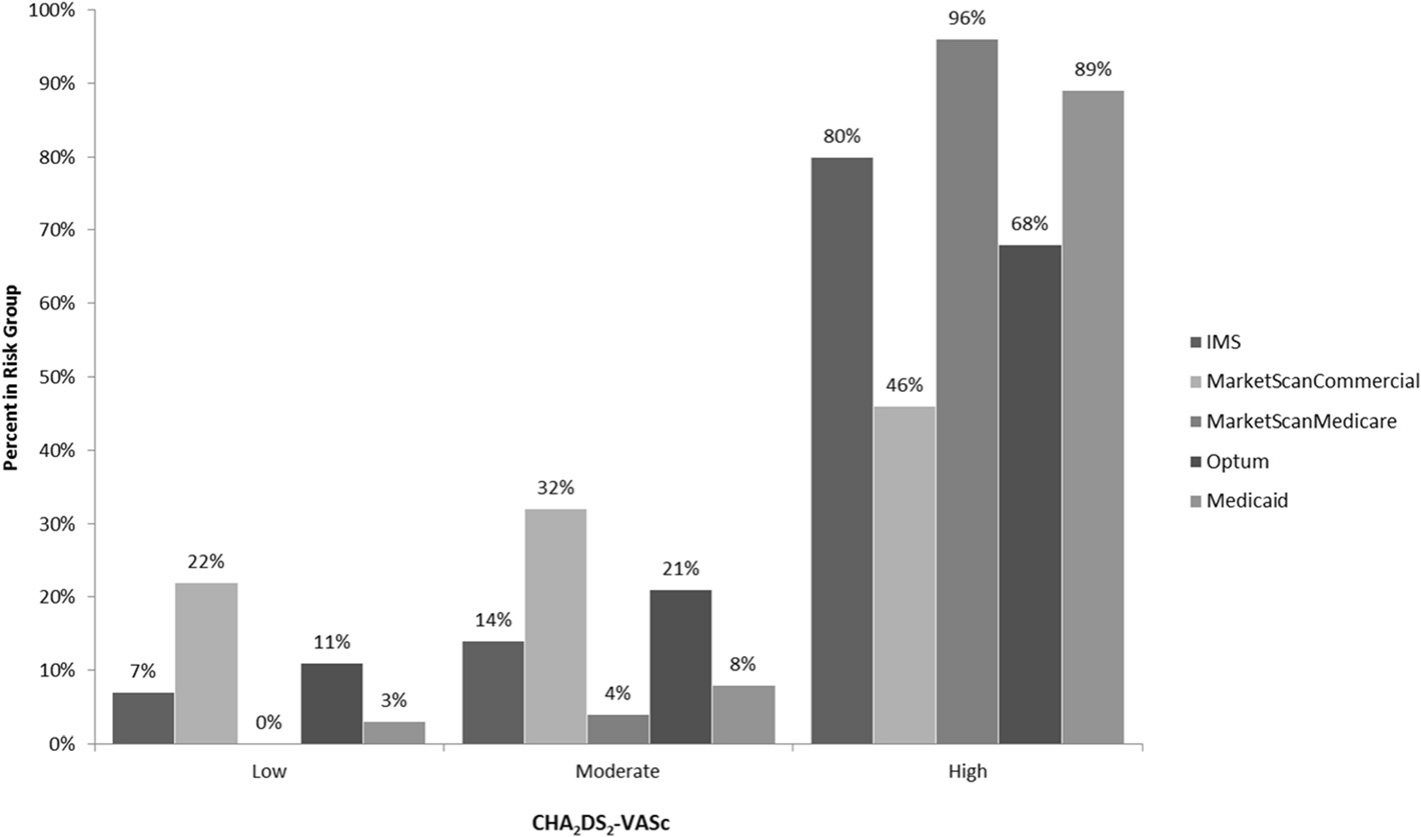
**Proportion of AF patients in each CHA**_**2**_**DS**_**2**_**-VASc stroke risk level.**

The number of patients meeting the cohort selection criteria varied across databases (Table 1); 30,757 IMS, 21,976 MarketScanCommercial, 38,643 MarketScanMedicare, 9,120 Optum, 4,901 Medicaid. Average age varied between 56 and 80 years, with MarketScanCommercial and Optum databases having generally younger populations. More than half of study patients were male, with the exception of the Medicaid database. Most patients were high risk, stratified according to CHADS_2_ or CHA_2_DS_2_-VASc scores (Figures 1, 2). In general, more than 50% of the patients had hypertension in the study period. Diabetes and coronary heart disease were other commonly observed conditions.

**Table 1:** Demographic and clinical characteristics

	**IMS**	**MarketScan Commercial**	**MarketScan Medicare**	**Optum**	**Medicaid**
**All patients, N**	30,757	21,976	38,643	9,120	4,901
**Demographic characteristics**					
Males (%)	69%	67%	53%	64%	39%
Average age (years)					
All	71.23	56.15	79.74	63.48	66.81
Female	73.95	55.92	81.03	65.30	69.14
Male	69.19	56.27	78.59	62.48	63.25
**Comorbidities (%)**					
Hypertension	62%	48%	60%	63%	69%
Diabetes	24%	22%	26%	25%	39%
Heart failure	26%	12%	28%	20%	33%
Acute myocardial infarction	6%	2%	3%	3%	3%
Coronary heart disease	34%	20%	35%	29%	39%
Other arrhythmias	26%	20%	26%	26%	24%

### Anticoagulation use in the study period

**Figure 3:**
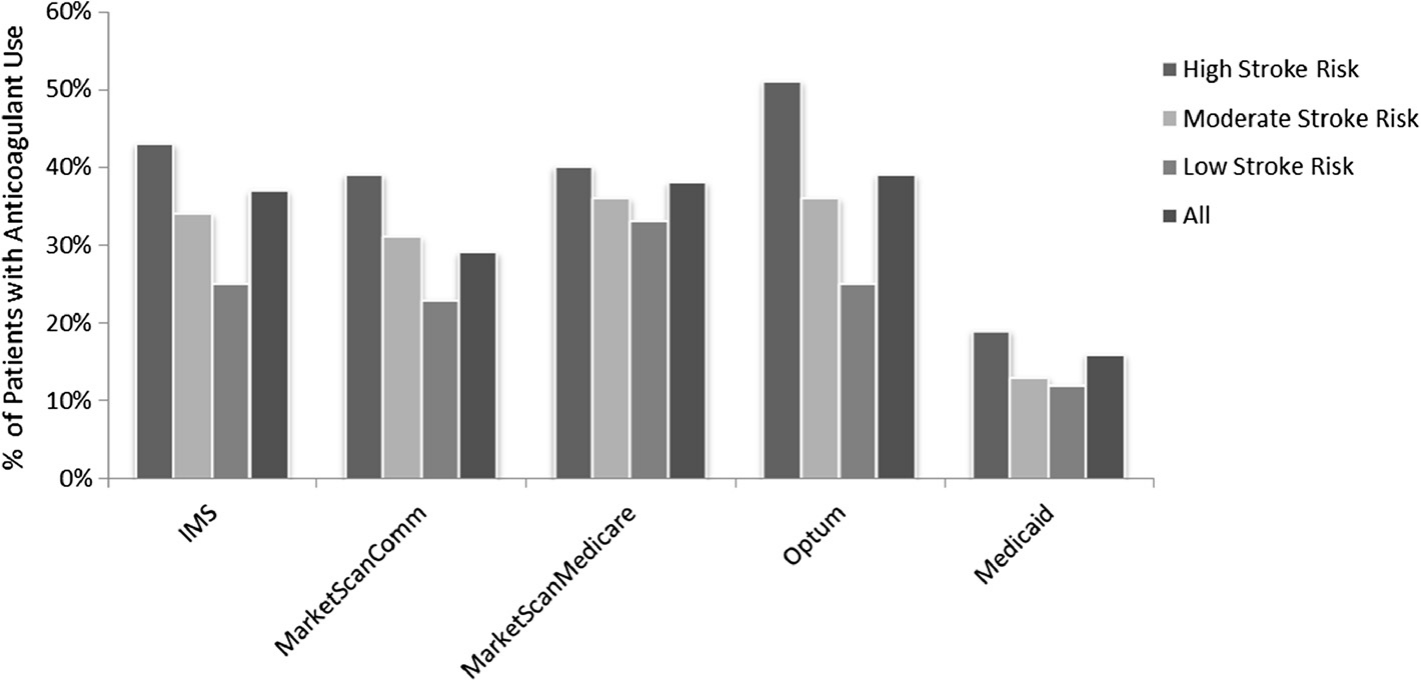
**Anticoagulant use among AF patients, stratified by CHADS**_**2 **_**stroke risk level.**

The overall percentage of patients receiving anticoagulants was less than 50% in all five databases (Table 2) ranging from 16%-39%; these rates were slightly higher among patients at high risk of stroke according to CHADS_2_ score (Figure 3). The anticoagulant MPR varied from 0.58 to 0.72 across all five databases. 29% to 59% of the patients had a gap in anticoagulant therapy. The time to first gap in anticoagulation therapy varied from 129 to 166 days across different databases.

**Table 2:** Anticoagulant use outcomes in the study period

	**IMS**	**MarketScan Commercial**	**MarketScan Medicare**	**Optum**	**Medicaid**
**All patients, N**	30,757	21,976	38,643	9,120	4,901
Patients receiving anticoagulant by stroke risk* and age category, N (%)					
All Stroke Risk Levels	11,382 (37%)	6,444 (29%)	14,686 (38%)	3,595 (39%)	803 (16%)
High Risk	6,832 (43%)	1,934 (39%)	9,822 (40%)	1,946 (51%)	566 (19%)
Age <65 years	1,252	1,877	58	672	476
Age: 65–74 years	1,484	57	1,651	386	38
Age ≥ 75 years	4,090	0	8,113	888	52
Moderate Risk	3,130 (34%)	2,415 (31%)	3,788 (36%)	1,091 (36%)	172 (13%)
Age <65 years	1,149	2,311	34	696	158
Age: 65–74 years	1,213	104	1,600	292	11
Age ≥ 75 years	764	0	2,154	103	3
Low Risk	1,420 (25%)	2,095 (23%)	1,076 (33%)	558 (25%)	65 (12%)
Age <65 years	813	1,940	12	423	63
Age: 65–74 years	603	155	1,064	135	2
Age ≥ 75 years	0	0	0	0	0
**Anticoagulant MPR**					
MPR	0.58	0.58	0.66	0.61	0.72
**Gap in Anticoagulation Therapy**					
Patients with a Gap in Anticoagulation Therapy, N(%)	6,745 (59%)	3,828 (59%)	6,687 (46%)	1,883 (52%)	233 (29%)
Average Time to First Gap in Anticoagulation Therapy (days)	132	150	157	144	129

MPR – Medication Possession Ratio.

*CHADS_2_ used to determine stroke risk.

### Stroke hospitalizations, anticoagulation use and bleeding events in the study period

During the study period, 1% to 2% of study patients experienced a stroke-related hospitalization (Table 3). Of those who were hospitalized, only 28% to 50% were treated with an anticoagulant in the outpatient setting prior to hospitalization. The percentage of patients with a bleeding event was low (≤1%) across all databases.

**Table 3:** Stroke-related hospitalizations, outpatient anticoagulant use and bleeding events among patients hospitalized for stroke in the study period

	**IMS**	**MarketScan Commercial**	**MarketScan Medicare**	**Optum**	**Medicaid**
**All patients, N**	30,757	21,976	38,643	9,120	4,901
**Patients with stroke-related hospitalizations**					
N (%)	478 (2%)	146 (1%)	912 (2%)	100 (1%)	40 (1%)
**Hospitalized stroke patients not treated with an anticoagulant in the outpatient setting**					
N (%)	276 (58%)	92 (63%)	571 (63%)	54 (54%)	20 (50%)
**Patients with bleeding event hospitalization**					
N (%)	222 (1%)	45 (0%)	258 (1%)	43 (0%)	30 (1%)
**Inpatient hospitalizations**					
N (%)	9,303 (30%)	4,500 (20%)	14,271 (37%)	2,361 (26%)	1,333 (27%)
**ER visits**					
N (%)	9,342 (30%)	4,836 (22%)	13,641 (35%)	2,311 (25%)	1,194 (24%)
**Outpatient visits**					
N (%)	27,322 (89%)	19,983 (91%)	33,854 (88%)	8,329 (91%)	3,805 (78%)

ER – emergency room.

### Other resource use (All-cause)

**Figure 4:**
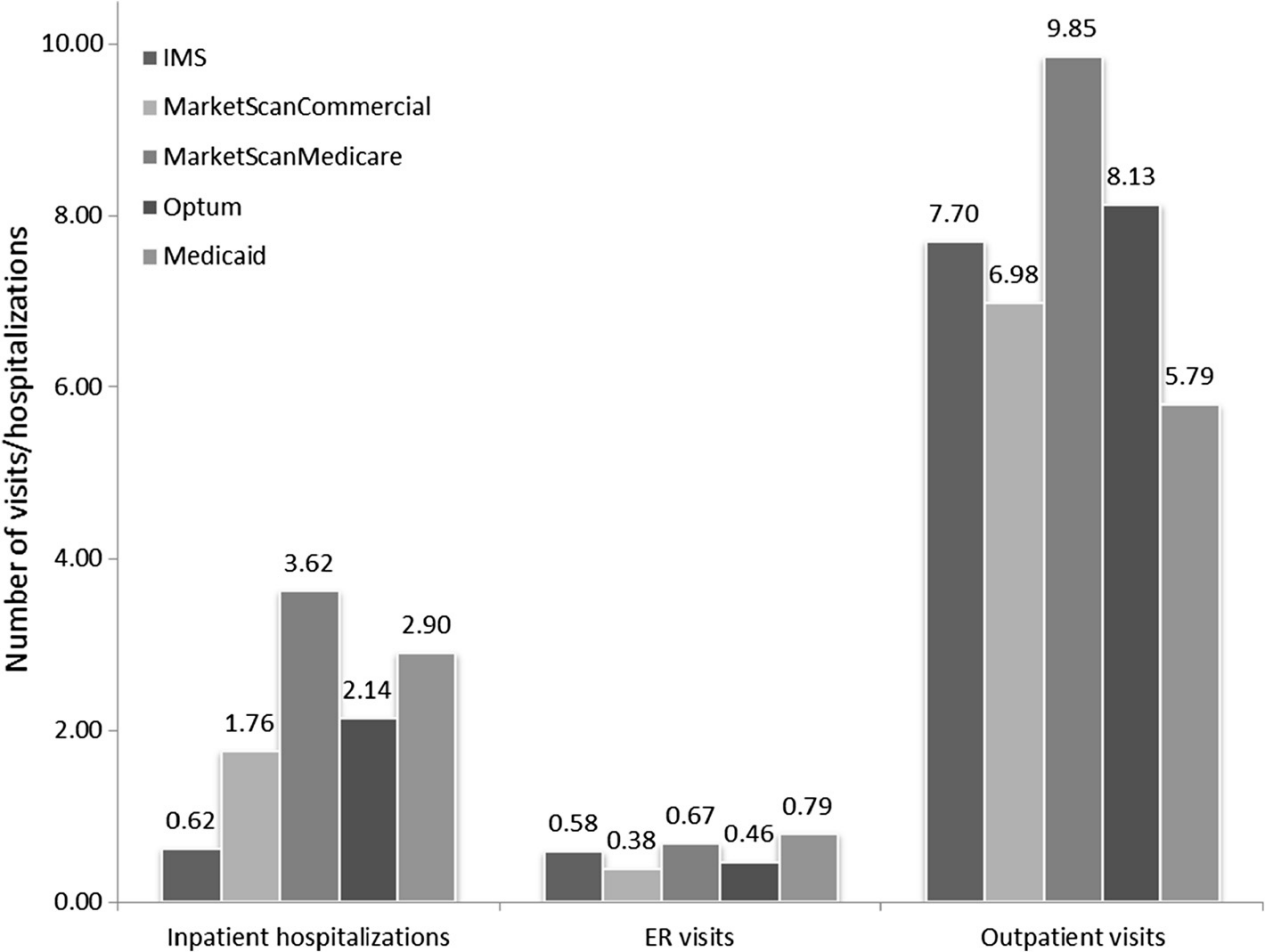
Mean all-cause resource use among AF patients during follow-up.

About 20% to 40% of the patients had an inpatient hospitalization or ER visit during the study period (Table 3). The average number inpatient hospitalizations varied from 0.62 to 3.62 across all databases (Figure 4). The average number of ER visits per patient was generally low (0.38 to 0.79 across all databases). Most of the patients had an outpatient visit and, on average, patients had more than six visits in the study period.

### INR outcomes

More than 55% of patients receiving warfarin had an INR test during the study period (Table 4). The average number of unique INRs per month of warfarin treatment and the monthly INR quality ratio varied from 0.51 to 2.05 and 0.30 to 0.64 across all databases, respectively.

**Table 4:** INR outcomes in the study period

	**IMS**	**MarketScan Commercial**	**MarketScan Medicare**	**Optum**	**Medicaid**
**All patients, N**	30,757	21,976	38,643	9,120	4,901
**INR frequency of occurrence: total warfarin patient population**					
N (%)	6,452/10,194 (63%)	5,536/6,372 (87%)	8,083/14,563 (56%)	2,002/3,159 (63%)	775/803 (97%)
**Average number of unique INRs per month of warfarin treatment**					
Unique INRs per month	0.72	1.02	0.51	0.62	2.05
**Monthly INR quality ratio**					
Quality ratio	0.38	0.53	0.30	0.36	0.64

INR – International Normalized Ratio.

## Discussion

### Summary

Condition-specific tools such as the one used for this analysis (AQuIA) offer an effective and easy way for increasing awareness with regards to effective management of AF. For example, the AQuIA is designed to interact with health plans to identify members with AF at high risk for stroke and providers that are not appropriately managing these patients based on guideline recommendations. Health plans can use the findings generated from the software tool to implement educational programs for patients and providers regarding the importance of stroke prevention among AF patients and recommendations regarding the need for anticoagulant prophylaxis. Findings from the software tool would be specific to the health plan’s patient population and consequently, provide more applicable data than general findings from published literature (e.g., registries).

Our analysis showed that the overall percentage of patients not receiving anticoagulants ranged from 61%-84% of the overall AF population and 49%-81% among high stroke risk patients. Anticoagulation MPR was generally low (0.58-0.72) with 29% to 59% of the patients having a gap in anticoagulation therapy and time to first gap occurring 129 to 166 days after starting an anticoagulant.

During the study period, 1% to 2% of study patients experienced a stroke-related hospitalization, and ≤1% a hospitalization for a bleeding event. Of those that were hospitalized for stroke, only 28% to 50% were treated with an anticoagulant in the outpatient setting prior to hospitalization. About 20% to 40% of the patients had any inpatient hospitalization or ER visit during the study period. Among patients receiving warfarin, 56% to 97% had an INR test during the study period.

### Comparison with other literature

Similar low anticoagulation utilization rates were reported in other studies using Medicaid and MarketScan data. Wess et al. [13] and Johnston et al. [17] reported that less than 12% (vs. 16% in our Medicaid population) of the Ohio Medicaid patients with AF were prescribed anticoagulants. Moreover, anticoagulation use among patients at high risk of stroke with atrial fibrillation/flutter was reported as 42.1% (vs. 39%-40% in our study for the commercial and Medicare supplemental populations, respectively), based on a study that used MarketScan data [14].

In addition, our findings were consistent with the literature on stroke risk and hospitalization rates among patients with AF. Boccuzzi et al. [18] analyzed a large cohort of commercially insured patients with AF and found 37.0% of the total population to have a CHADS_2_ score ≥ 2, indicating high stroke risk. Among patients receiving an anticoagulant, we found a similar rate, with an average of 43% of commercially insured patients with a CHADS_2_ score ≥ 2. Boccuzzi et al. also reported a bleed rate of approximately 6% over a one-year study period. This is higher than the bleed rates found in our study (1%); however, the study conducted by Boccuzzi and colleagues included several additional ICD-9-CM codes for the identification of bleeding events [18]. A recent study by Naccarelli et al. using the MarketScan Medicare Supplemental database found that 33.5% of AF patients were hospitalized for any reason (vs. 37% in our study), approximately 2% for a stroke or transient ischemic attack (vs. 2% in our study), and approximately 1% for a bleeding event (vs. 1% in our study) during the first year of follow-up [19].

### Implications of our work

Current medical guidelines for stroke prevention recommend that all patients with AF who are at high risk for stroke receive prophylaxis, unless contraindicated [12,20]. Appropriate thromboprophylaxis has been shown to reduce the annual incidence of stroke in AF patients by more than 60% [6]. In our analysis, 49%-81% of patients at high risk for stroke did not receive anticoagulation, with particularly high proportions in the Medicaid population (81%). These levels are much higher than published literature, which has shown that appropriate anticoagulation rates of high risk patients as high as 80% are attainable [9]. This demonstrates how practice patterns can differ by payer type; a finding which could be taken into consideration when designing AF disease management programs.

In addition to appropriate thromboprophylaxis, optimal management of AF involves proper risk identification and adequate INR monitoring. From a physician perspective, stroke risk stratification emerges as a challenge as more than 12 risk stratification schemes have been proposed [21]. Moreover, there is reluctance among physicians to prescribe anticoagulants in the elderly (due to cognitive and physical impairment concerns) [22], as well as for those with perceived bleeding risk or history of falls [23]. Incomplete knowledge of guidelines also contribute to sub-optimal management of AF [24]. From the patients’ perspective, poor adherence, concerns about potential adverse events and perceived lifestyle restrictions associated with anticoagulants, and limited knowledge about AF related stroke risk and benefits of anticoagulation are potential barriers to effective disease management [25]. Findings from our analysis may demonstrate this reluctance to prescribe anticoagulants for reasons not identifiable in claims data. Therefore, it becomes important for payers to discuss with physicians the reasons for non-treatment among eligible high risk AF patients if a decline in anticoagulant prophylaxis in the population is observed over time.

### Limitations

This study was designed as a descriptive analysis and was not intended to investigate outcomes from different databases. Techniques such as propensity score matching were not employed. Dispersion around the mean values was not evaluated (e.g., using standard deviation or ranges). These analyses relied on claims data, which are used primarily for administrative (i.e., billing and operations) purposes and therefore do not reflect all clinical variables that are taken into account by physicians when making treatment decisions. INRs in our study reflect only those captured in claims data. INRs taken during office visits and not recorded on a claim, or those recorded by patients using home kits are not captured in this analysis, potentially underestimating INR use. The limited data on INR use may also have an impact on the interpretation of treatment pattern results, particularly adherence to anticoagulants, where changes in dosing are dependent on the results of laboratory monitoring. INR testing during observed gaps in therapy, if available, would potentially indicate ongoing anticoagulant use.

Patients contraindicated for anticoagulant use (e.g., patients at high risk of bleeding) were not excluded, potentially resulting in an overestimate of the extent to which anticoagulants are not properly utilized. Furthermore, continuous patient eligibility was not required to be included in the study, potentially resulting in underestimation of events and resource use. Lastly, we did not assess the use of over-the-counter medication such as aspirin.

## Conclusions

The AQuIA provided a consistent platform for analysis of treatment patterns across multiple AF populations with varying baseline characteristics. Results from the analyzer show that many AF patients in selected commercial, Medicare-eligible, and Medicaid populations, including those at high risk of stroke, do not receive appropriate thromboprophylaxis, as recommended by treatment guidelines. Further investigation of the impact of this treatment pattern on patient outcomes, such as the direct relationship between low levels of treatment and stroke incidence rates among multiple payer populations, is warranted. Increased use of the analyzer and similar software may support enhanced education efforts aimed at improving adherence to guidelines and quality of care.

## Competing interests

AAP, BM, WN, SM, and JS are employees of the study sponsor, Janssen Scientific Affairs, LLC, and are Johnson & Johnson stockholders. KL, DB, GO, and JM have no disclosures to report.

## Authors’ contributions

KL and JM contributed to the concept and design of the study, interpretation of data, and provided critical revisions to the manuscript. DB analyzed the data and contributed to the drafting of the manuscript. AAP contributed to the concept and design of the study, interpretation of data, and the drafting of the manuscript. BM, WM, SM, JS and GO contributed to the concept and design of the study and the drafting of the manuscript. All authors read and approved the final manuscript.

## Pre-publication history

The pre-publication history for this paper can be accessed here:

http://www.biomedcentral.com/1472-6963/14/329/prepub

## Supplementary Material

Additional file 1: Table S1 ICD-9-CM codes for CHADS2 and CHA2DS2-VASc Conditions. Click here for file

Additional file 2: Table S2 ICD-9-CM codes for comorbid conditions. Click here for file

Additional file 3: Table S3 ICD-9-CM codes for complications of interest. Click here for file
