# Supplementary material for: Anticoagulant use for the prevention of stroke in patients with atrial fibrillation: findings from a multi-payer analysis
Source: BMC Health Serv Res. 2014 Jul 28;14:329. doi: 10.1186/1472-6963-14-329 (PMC4126814; doi:10.1186/1472-6963-14-329)
Supplement: Additional file 1: Table S1 — ICD-9-CM codes for CHADS2 and CHA2DS2-VASc Conditions. [file 1472-6963-14-329-S1.doc]

Additional file 1: Table S1. ICD-9-CM codes for CHADS2 and CHA2DS2-VASc Conditions

| **Diagnosis** | **ICD-9-CM Diagnosis Codes** |
| --- | --- |
| Ischemic and/or hemorrhagic stroke | 430 Subarachnoid hemorrhage  431 Intracerebral hemorrhage  432.x Other and unspecified intracranial hemorrhage  433.01 Occlusion and stenosis of basilar artery with cerebral infarction  433.11 Occlusion and stenosis of carotid artery with cerebral infarction  433.21 Occlusion and stenosis of vertebral artery with cerebral infarction  433.31 Occlusion and stenosis of multiple and bilateral precerebral arteries with cerebral infarction  433.81 Occlusion and stenosis of other specified precerebral artery with cerebral infarction  433.91 Occlusion and stenosis of unspecified precerebral artery with cerebral infarction  433.xx Occlusion and stenosis of precerebral arteries  434.01 Cerebral thrombosis with cerebral infarction  434.11 Cerebral embolism with cerebral infarction  434.91 Unspecified cerebral artery occlusion with cerebral infarction  437.1 Other generalized ischemic cerebrovascular disease  437.3 Cerebral aneurysm, nonruptured  997.02 Iatrogenic cerebrovascular infarction or hemorrhage |
| Transient ischemia attack | 435.xx Transient cerebral ischemia |
| Hypertension | 401.x Essential hypertension  405.xx Secondary hypertension |
| Diabetes | 250.xx Diabetes mellitus  249.xx Secondary diabetes mellitus |
| Heart failure  Left ventricular dysfunction | 428.xx Heart failure  429.9 Heart disease unspecified (eg, ventricular dysfunction) |
| | Acute myocardial  infarction (heart attack) | 410.xx Acute myocardial infarction | | --- | --- | | 410.xx Acute myocardial infarction |
| Coronary heart disease | 414.0 Coronary atherosclerosis  414.9 Unspecified chronic ischemic heart disease  414.xx Other forms of chronic ischemic heart disease |
| Peripheral artery disease | 443.9 Peripheral artery disease |
| Aortic plaque | 440.0 Artherosclerosis of aorta |
| Thromboembolism | **Deep vein thromboembolism:**  451.1x Phlebitis and thrombophlebitis of deep vessels of lower extremities  451.2 Phlebitis and thrombophlebitis of lower extremities, unspecified  453.4x Other venous embolism and thrombosis; venous embolism and thrombosis of deep vessels of lower extremity  453.5x Chronic venous embolism and thrombosis of unspecified deep vessels of lower extremity  453.8 Other venous embolism and thrombosis of other specified veins  453.9 Other venous embolism and thrombosis of unspecified site  **Pulmonary embolism:**  415.1x Pulmonary embolism and infarction  **Obstetrics-related embolism codes:**  639.6 Embolism  673.20 Obstetrical blood-clot embolism, unspecified as to episode of care  673.21 Obstetrical blood-clot embolism, with delivery, with or without mention of antepartum condition  673.22 Obstetrical blood-clot embolism, with mention of postpartum complication  673.23 Obstetrical blood-clot embolism, antepartum  673.24 Obstetrical blood-clot embolism, postpartum  634.60 Unspecified spontaneous abortion complicated by embolism  634.61 Incomplete spontaneous abortion complicated by embolism  634.62 Complete spontaneous abortion complicated by embolism  635.60 Unspecified legally induced abortion complicated by embolism  635.61 Incomplete legally induced abortion complicated by embolism  635.62 Complete legally induced abortion complicated by embolism  636.60 Unspecified illegally induced abortion complicated by embolism  636.61 Incomplete illegally induced abortion complicated by embolism  636.62 Complete illegally induced abortion complicated by embolism  637.60 Abortion, unspecified as to completion or legality, complicated by embolism  637.61 Legally unspecified abortion, incomplete, complicated by embolism  637.62 Legally unspecified abortion, complete, complicated by embolism  638.6 Failed attempted abortion complicated by embolism  639.6 Embolism following abortion or ectopic and molar pregnancies  673.80 Other obstetrical pulmonary embolism, unspecified as to episode of care  673.81 Other obstetrical pulmonary embolism, with delivery, with or without mention of antepartum condition  673.82 Other obstetrical pulmonary embolism, with delivery, with mention of postpartum complication  673.83 Other obstetrical pulmonary embolism, antepartum  673.84 Other obstetrical pulmonary embolism, postpartum |
