# Supplementary material for: Anticoagulant use for the prevention of stroke in patients with atrial fibrillation: findings from a multi-payer analysis
Source: BMC Health Serv Res. 2014 Jul 28;14:329. doi: 10.1186/1472-6963-14-329 (PMC4126814; doi:10.1186/1472-6963-14-329)
Supplement: Additional file 2: Table S2 — ICD-9-CM codes for comorbid conditions. [file 1472-6963-14-329-S2.doc]

Additional file 2: Table S2. ICD-9-CM codes comorbid conditions

| **Diagnosis** | **ICD-9-CM Diagnosis Codes** |
| --- | --- |
| Depressive Disorders | 296.2x Major depressive disorder, single episode  296.3x Major depressive disorder, recurrent episode  296.5x Bipolar I disorder, most recent episode (or current) depressed  296.82 Atypical depressive disorder  311 Depressive disorder, not elsewhere classified |
| Ischemic and/or  hemorrhagic stroke | 430 Subarachnoid hemorrhage  431 Intercerebral hemorrhage  432.x Other and unspecified intracranial hemorrhage  433.xx Occlusion and stenosis of precerebral arteries  434.01 Cerebral thrombosis with cerebral infarction  434.11 Cerebral embolism with cerebral infarction  434.91 Unspecified cerebral artery occlusion with cerebral infarction  437.1 Other generalized ischemic cerebrovascular disease  437.3 Cerebral aneurysm, nonruptured  997.02 Iatrogenic cerebrovascular infarction or hemorrhage |
| Hypertension | 401.x Essential hypertension  405.xx Secondary hypertension |
| General anxiety disorder | 300.02 Generalized anxiety disorder |
| Diabetes | 250.xx Diabetes mellitus  249.xx Secondary diabetes mellitus |
| Heart Failure | 428.xx Heart failure |
| Acute myocardial infarction  (heart attack) | 410.xx Acute myocardial infarction |
| Coronary heart disease | 414.0 Coronary atherosclerosis  414.9 Unspecified chronic ischemic heart disease  414.xx Other forms of chronic ischemic heart disease |
| COPD/bronchitis | 491.20 Obstructive chronic bronchitis without exacerbation  491.21 Obstructive chronic bronchitis with (acute) exacerbation  491.22 Obstructive chronic bronchitis with acute bronchitis  492 Emphysema  518.1 Interstitial emphysema  518.2 Compensatory emphysema |
| Arrhythmias other than  atrial fibrillation | 426.0x-426.6x  426.82 Long QT syndrome  427.0x Tachycardia, paroxysmal supraventricular  427.4x Ventricular fibrillation and flutter  427.6x Premature beats, unspecified  427.8x Other specified cardiac dysrhythmias  427.9x Cardiac dysrhythmia, unspecified |
