# Supplementary material for: Anticoagulant use for the prevention of stroke in patients with atrial fibrillation: findings from a multi-payer analysis
Source: BMC Health Serv Res. 2014 Jul 28;14:329. doi: 10.1186/1472-6963-14-329 (PMC4126814; doi:10.1186/1472-6963-14-329)
Supplement: Additional file 3: Table S3 — ICD-9-CM codes for complications of interest. [file 1472-6963-14-329-S3.doc]

Additional file 3: Table S3. ICD-9-CM codes for complications of interest

| **Diagnosis** | **ICD-9-CM Diagnosis Codes** |
| --- | --- |
| Ischemic stroke | 433.01 Occlusion and stenosis of basilar artery with cerebral infarction  433.11 Occlusion and stenosis of carotid artery with cerebral infarction  433.21 Occlusion and stenosis of vertebral artery with cerebral infarction  433.31 Occlusion and stenosis of multiple and bilateral precerebral arteries with cerebral infarction  433.81 Occlusion and stenosis of other specified precerebral artery with cerebral infarction  433.91 Occlusion and stenosis of unspecified precerebral artery with cerebral infarction  434.01 Cerebral thrombosis with cerebral infarction  434.11 Cerebral embolism with cerebral infarction  434.91 Unspecified cerebral artery occlusion with cerebral infarction  437.1 Other generalized ischemic cerebrovascular disease  437.3 Cerebral aneurysm, nonruptured  997.02 Iatrogenic cerebrovascular infarction or hemorrhage |
| Hemorrhagic stroke | 430 Subarachnoid hemorrhage  431 Intracerebral hemorrhage  432.x Other and unspecified intracranial hemorrhage  997.02 Iatrogenic cerebrovascular infarction or hemorrhage |
| Ischemic and/or  hemorrhagic stroke | 430 Subarachnoid hemorrhage  431 Intracerebral hemorrhage  432.x Other and unspecified intracranial hemorrhage  433.xx Occlusion and stenosis of precerebral arteries  434.01 Cerebral thrombosis with cerebral infarction  434.11 Cerebral embolism with cerebral infarction  434.91 Unspecified cerebral artery occlusion with cerebral infarction  437.1 Other generalized ischemic cerebrovascular disease  437.3 Cerebral aneurysm, nonruptured  997.02 Iatrogenic cerebrovascular infarction or hemorrhage |
| Major bleed | 423.00 Hemopericardium  430 Subarachnoid hemorrhage  431 Intracerebral hemorrhage  432.x Other and unspecified intracranial hemorrhage  455.20 Hemorrhoids, internal with complication  455.5 Hemorrhoids, external with complication  455.8 Hemorrhoids, NOS with complication  456.0 Esophageal varices with bleeding  456.20 Esophageal varices in other diseases  459.0 Hemorrhage NOS  530.70 Mallory-Weiss Syndrome  530.80 Other specified disorders of esophagus  530.82 Esophageal hemorrhage  531.00 Acute gastric ulcer with hemorrhage  531.01 Acute gastric ulcer with hemorrhage with obstruction  531.20 Acute gastric ulcer with hemorrhage and perforation  531.21 Acute gastric ulcer with hemorrhage and perforation with obstruction  531.40 Chronic or unspecified gastric ulcer with hemorrhage  531.41 Chronic or unspecified gastric ulcer with hemorrhage with obstruction  531.60 Chronic or unspecified gastric ulcer with hemorrhage and perforation  531.61 Chronic or unspecified gastric ulcer with hemorrhage and perforation with obstruction  532.00 Acute duodenal ulcer with hemorrhage  532.21 Acute duodenal ulcer with hemorrhage and perforation with obstruction  532.40 Chronic or unspecified duodenal ulcer with hemorrhage  532.41 Chronic or unspecified duodenal ulcer with hemorrhage with obstruction  532.60 Chronic or unspecified duodenal ulcer with hemorrhage and perforation without obstruction  532.61 Chronic or unspecified duodenal ulcer with hemorrhage and perforation with obstruction  533.00 Acute peptic ulcer, unspecified site, with hemorrhage  533.01 Acute peptic ulcer, unspecified site, with hemorrhage with obstruction  533.20 Acute peptic ulcer, unspecified site, with hemorrhage and perforation without obstruction  533.21 Acute peptic ulcer, unspecified site, with hemorrhage and perforation with obstruction  533.40 Chronic or unspecified peptic ulcer, unspecified site, with hemorrhage  533.41 Chronic or unspecified peptic ulcer, unspecified site, with hemorrhage with obstruction  533.60 Chronic or unspecified peptic ulcer, unspecified site, with hemorrhage and perforation without obstruction  533.61 Chronic or unspecified peptic ulcer, unspecified site, with hemorrhage and perforation with obstruction  534.00 Acute gastrojejunal ulcer with hemorrhage without obstruction  534.01 Acute gastrojejunal ulcer with hemorrhage with obstruction  534.20 Acute gastrojejunal ulcer with hemorrhage and perforation without obstruction  534.21 Acute gastrojejunal ulcer with hemorrhage and perforation with obstruction  534.40 Chronic or unspecified gatrojejunal ulcer with hemorrhage without obstruction  534.41 Chronic or unspecified gatrojejunal ulcer with hemorrhage with obstruction  534.60 Chronic or unspecified gatrojejunal ulcer with hemorrhage and perforation without obstruction  534.61 Chronic or unspecified gatrojejunal ulcer with hemorrhage and perforation with obstruction  535.01 Acute gastritis with hemorrhage  535.11 Atrophic gastritis with hemorrhage  535.21 Gastric mucosal hypertrophy with hemorrhage  535.31 Alcoholic gastritis with hemorrhage  535.41 Other specified gastritis with hemorrhage  535.51 Unspecified gastritis and gastroduodentis with hemorrhage  535.61 Duodentis with hemorrhage  537.80 Other specified disorders of stomach and duodenum  562.02 Diverticulosis of small intestine with hemorrhage  562.03 Diverticulitis of small intestine with hemorrhage  562.12 Diverticulosis of colon with hemorrhage  562.13 Diverticulitis of colon with hemorrhage  568.81 Hemoperitoneum (nontraumatic)  569.30 Hemorrhage of rectum and anus  569.85 Angiodysplasia of intestine with hemorrhage  578.00 Hematemesis  578.10 Blood in stool  578.90 Hemorrhage of gastrointestinal tract, unspecified  593.81 Vascular disorders of kidney  596.7 Hemorrhage into bladder wall  599.70 Hematuria  623.80 Other specified noninflammatory disorders of vagina  626.20 Excessive or frequent menstruation; Abnormally heavy menstrual bleeding  626.60 Metrorrhagia  719.10 Hemarthrosis site unspecified  719.11 Hemarthrosis involving shoulder region  719.12 Hemarthrosis involving upper arm  719.13 Hemarthrosis involving forearm  719.14 Hemarthrosis involving hand  719.15 Hemarthrosis involving pelvic region and thigh  719.16 Hemarthrosis involving lower leg  719.17 Hemarthrosis involving ankle and foot  719.18 Hemarthrosis involving other specified sites  784.70 Epistaxis  784.80 Hemorrhage from throat  786.3 Hemoptysis  719.14 Hemarthrosis involving hand  719.15 Hemarthrosis involving pelvic region and thigh  719.16 Hemarthrosis involving lower leg  719.17 Hemarthrosis involving ankle and foot  719.18 Hemarthrosis involving other specified sites  784.70 Epistaxis  784.80 Hemorrhage from throat  786.3 Hemoptysis |
